# Spatial, Ecologic, and Clinical Epidemiology of Community-Onset, Ceftriaxone-Resistant *Enterobacteriaceae*, Cook County, Illinois, USA

**DOI:** 10.3201/eid2708.204235

**Published:** 2021-08

**Authors:** Vanessa Sardá, William E. Trick, Huiyuan Zhang, David N. Schwartz

**Affiliations:** Cook County Health, Chicago, IL, USA (V. Sardá, W.E. Trick, H. Zhang, D.N. Schwartz);; Rush Medical College, Chicago (V. Sardá, W.E. Trick, D.N. Schwartz)

**Keywords:** *Enterobacteriaceae*, *Escherichia coli*, ceftriaxone-resistance, antimicrobial resistance, bacteria, bacterial infections, community-onset, Cook County, Illinois, United States

## Abstract

We performed a spatial and mixed ecologic study of community-onset *Enterobacteriaceae* isolates collected from a public healthcare system in Cook County, Illinois, USA. Individual-level data were collected from the electronic medical record and census tract–level data from the US Census Bureau. Associations between individual- and population-level characteristics and presence of ceftriaxone resistance were determined by logistic regression analysis. Spatial analysis confirmed nonrandom distribution of ceftriaxone resistance across census tracts, which was associated with higher percentages of Hispanic, foreign-born, and uninsured residents. Individual-level analysis showed that ceftriaxone resistance was associated with male sex, an age range of 35–85 years, race or ethnicity other than non-Hispanic Black, inpatient encounter, and percentage of foreign-born residents in the census tract of isolate provenance. Our findings suggest that the likelihood of community-onset ceftriaxone resistance in *Enterobacteriaceae* is influenced by geographic and population-level variables. The development of effective mitigation strategies might depend on better accounting for these factors.

The continuous rise of infections secondary to extended-spectrum beta-lactamase (ESBL)–producing *Enterobacteriaceae* in the United States is a complex public health problem and considered a serious threat by the Centers for Disease Control and Prevention (*1*). Recently, the incidence of infections caused by ESBL producers in the United States was noted to have increased by 53.3% during 2019–2017, driven largely by a surge in community-onset cases (*2*). Globally, a similar trend has been described, and developing countries bear a disproportionate burden of infections secondary to these drug-resistant pathogens (*3*–*5*). The steady increases in rates of infections caused by ESBL-producing *Escherichia coli* and *Klebsiella pneumoniae* persist despite antimicrobial stewardship and infection control efforts (*6*,*7*).

Initially confined to the healthcare environment, infections caused by ESBL-producing *Enterobacteriaceae* among patients without previous healthcare exposure have been described since the mid-2000s (*8*,*9*). This epidemiologic shift has been largely attributed to the emergence of the CTX-M–producing *E. coli* sequence type (ST) 131 clone, which expanded rapidly throughout the United States and remains the most prevalent ESBL-producing *E. coli* clone in the community (*10*). In addition to higher virulence and transmissibility of the *E. coli* ST131 clone, its therapeutic management is particularly challenging because of its associated resistance to commonly used oral antimicrobial drugs such as quinolones and trimethoprim/sulfamethoxazole (*6*,*10*).

From an epidemiologic standpoint, multiple transmission pathways for community-onset ESBL-producing *Enterobacteriaceae* have been proposed. Potential sources of acquisition outside of healthcare environments include gastrointestinal colonization after international travel (*11*,*12*) and transmission among household members (*7*,*13*). In addition, ESBL-producing *Enterobacteriaceae* have been isolated from foodstuffs (*14*,*15*), livestock (*14*), and waterways (*16*,*17*), all of which have been posited as potential sources for human colonization and subsequent infection. A better understanding of the epidemiology of community-onset infections caused by ESBL-producing bacteria across geographic areas can help identify areas with higher disease burden and suggest pathways of transmission and mitigation strategies that are potentially unique to each region. Spatial and ecologic analyses help to address the influence of geography and population-level variables on disease distribution in a given region.

We conducted an epidemiologic analysis of the distribution of community-onset, ceftriaxone-resistant (CTX-R) *Enterobacteriaceae* from a single healthcare system in Cook County, Illinois, USA. We hypothesized that population-level characteristics are contributing factors for the presence of CTX-R *Enterobacteriaceae* in a geographic area and at the individual level.

## Methods

Cook County Health (CCH) is a large safety-net healthcare system in Chicago and suburban Cook County, Illinois. It consists of a 450-bed teaching hospital near downtown Chicago, a small community hospital in the South Side of Chicago, a small hospital and clinic for the treatment of detainees in the Cook County jail, and 13 community clinics distributed throughout Cook County. In 2018, CCH cared for 205,322 persons, most of whom self-identified as non-Hispanic Black (49.1%) or Hispanic (32.7%). Through electronic queries, we identified all culture isolates of the commonest *Enterobacteriaceae* species collected at CCH: *E. coli*, *K. pneumoniae*, *Enterobacter cloacae*, *Proteus mirabilis*, *Enterobacter aerogenes*, and *Klebsiella oxytoca* collected from Cook County residents during January 1, 2016–December 31, 2018. We determined antimicrobial susceptibilities by using the MicroScan Gram-negative panel (Beckman Coulter, https://www.beckmancoulter.com) and interpreted results by using Clinical and Laboratory Standards Institute breakpoints (*18*). We obtained antimicrobial susceptibilities retrospectively and did not retain any isolates for further analysis. We excluded isolates collected from persons <18 years of age, surveillance isolates, isolates with intermediate susceptibility to ceftriaxone or intermediate susceptibility or resistance to carbapenems, and duplicate isolates (defined as isolates from the same persons, of the same species, and collected within 30 days). To select for community-onset isolates, we included only isolates collected in the ambulatory clinic or emergency department (ED) setting and those collected during the first 2 days of hospitalization.

Demographic characteristics, collected from the electronic medical record (EMR), were patient sex and age and self-identified race and ethnicity, categorized as non-Hispanic Black, non-Hispanic White, Hispanic, or other. We classified encounter types as outpatient (ambulatory clinic), ED, or inpatient. Census-tract variables for Cook County were obtained from the 2017 US Census Bureau American Community Survey 5-year estimates (*19*). We extracted census tract data on race and ethnicity, immigration status (US-born or foreign-born), deprivation (households below poverty level and uninsured status), and overcrowding (>1.5 occupants per room).

### Spatial Analysis

Cook County, which includes the city of Chicago, contains 1,319 land census tracts and has an estimated population of 5,149,580 residents (*19*). We used ArcGIS version 10.4.1 (ESRI, https://www.esri.com) to geocode isolates to their census tract of provenance by using residential addresses available in the EMR. We calculated and mapped the percentage of CTX-R isolates in each census tract (i.e., the number of CTX-R isolates divided by the number of all isolates multiplied by 100). To minimize imprecision of CTX-R percentages in census tracts with low number of isolates, we excluded from the spatial analysis census tracts that had <3 isolates collected during the study period. We used spatial autocorrelation analysis (Moran I) to identify whether *Enterobacteriaceae* CTX-R percentages were distributed at random or clustered in census tracts across Cook County. Similarly, we conducted spatial autocorrelation analysis on CTX-R percentage distribution of *E. coli* isolates alone.

### Ecologic Analysis

After excluding census tracts with <3 isolates, we categorized the remaining census tracts on the basis of the presence or absence of a CTX-R isolate. We evaluated the relationship between each population-level variable and the presence of >1 CTX-R isolates in a census tract by using bivariate logistic regression, summarized by odds ratios (ORs) and corresponding 95% CIs. We conducted a similar analysis for *E. coli* isolates alone.

### Individual Risk Analysis

We categorized individual *Enterobacteriaceae* isolates on the basis of the identification of ceftriaxone resistance in the susceptibility panel. We included all isolates in the analysis of individual risk. The variables of interest were the individual demographic variables collected from the EMR and the type of clinical encounter. In addition, we included an ecologic variable, the percentage of foreign-born population in the census tract of residency. We evaluated the relationship between each variable and identification of ceftriaxone resistance in an individual isolate by using bivariate logistic regression, summarized by ORs and corresponding 95% CIs. We conducted all statistical analyses by using Stata version 14.2 (StataCorp, https://www.stata.com).

## Results

We collected 12,892 *Enterobacteriaceae* isolates at CCH during the study period, 10,891 of which met the inclusion criteria and were included in the dataset. We summarized the demographic and clinical characteristics of the patients from whom *Enterobacteriaceae* isolates were collected (Table 1). Most isolates were collected from women (7,853 [72.1%]), were from urine specimens (9,315 [85.5%]), were collected in ambulatory clinics (5,889 [54.1%]), or were identified as *E. coli* (7,977 [73.2%]). A total of 1,035 (9.5%) *Enterobacteriaceae* (817 [10.2%] *E. coli* isolates) were resistant to ceftriaxone. We observed no notable trends in ceftriaxone resistance across study years.

**Table 1:** Demographic and clinical characteristics of patients from whom selected *Enterobacteriaceae* isolates were collected, Cook County Health healthcare system, Illinois, USA, 2016–2018

Characteristic	No. isolates (%)
Total no. isolates	10,891 (100)
Sex	
F	7,853 (72.1)
M	3,038 (27.9)
Age group, y	
18–34	2,011 (18.6)
35–51	3,109 (28.5)
52–68	4,092 (37.5)
69–85	1,471 (13.5)
>85	208 (1.9)
Race and ethnicity	
Non-Hispanic White	997 (9.2)
Non-Hispanic Black	4,394 (40.4)
Hispanic	4,898 (44.9)
Other	602 (5.5)
Encounter type	
Outpatient	5,889 (54.1)
Emergency department	2,890 (26.5)
Inpatient	2,112 (19.4)
Organism	
* Escherichia coli*	7,977 (73.2)
* Klebsiella pneumoniae*	1,367 (12.6)
* Proteus mirabilis*	811 (7.5)
* Enterobacter cloacae*	376 (3.4)
* Klebsiella oxytoca*	197 (1.8)
* Enterobacter aerogenes*	163 (1.5)
Specimen type	
Urine	9,315 (85.5)
Wound	981 (9.0)
Blood	384 (3.6)
Other	211 (1.9)
Ceftriaxone susceptibility	
Susceptible	9,856 (90.5)
Resistant	1,035 (9.5)

**Figure 1:**
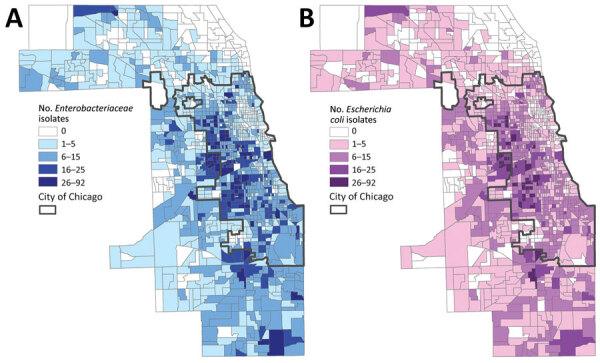
Number of *Enterobacteriaceae* (A) and *Escherichia coli* (B) isolates collected from patients in the Cook County Health healthcare system, by Cook County census tract, Illinois, USA, 2016–2018.

In the 1,319 land census tracts in Cook County, we collected *Enterobacteriaceae* isolates from residents of 1,131 (85.8%) and *E. coli* alone from residents of 1,085 (82.3%). The mean number of such isolates per census tract was 9.6 (SD + 9.28, range 1–92), and the mean number of *E. coli* isolates obtained per census tract was 7.4 (SD + 7.16, range 1–62). We plotted choropleth maps depicting the geographic distribution of all *Enterobacteriaceae* isolates and *E. coli* isolates alone (Figure 1). Among census tracts from which >1 isolate was obtained, CTX-R *Enterobacteriaceae* isolates were identified in 500 (44.2%), and most CTX-R isolates (561 [54.2%]) came from only 125 (11%) census tracts. In the case of CTX-R *E. coli* isolates, 424 (39.1%) of the 1,085 census tracts had a CTX-R *E. coli* isolate reported during the study period, and only 93 (8.6%) census tracts accounted for 406 (49.7%) of all CTX-R *E. coli* isolates.

**Figure 2:**
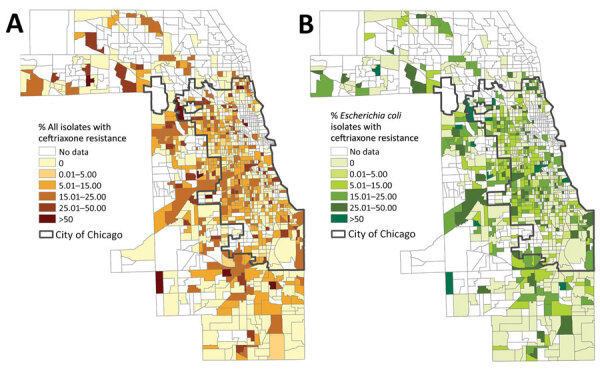
Percentage of ceftriaxone-resistant *Enterobacteriaceae* (A) and *Escherichia coli* (B) isolates collected from patients in the Cook County Health healthcare system, by Cook County census tract, Illinois, USA, 2016–2018.

A total of 886 census tracts had >3 *Enterobacteriaceae* isolates collected during the study period and were included in the spatial and ecologic analyses. The mean CTX-R percentage among these census tracts was 8.7%. Autocorrelation analysis (Moran I) indicated that CTX-R percentages among all isolates were not distributed randomly across Cook County census tracts (index 0.02, p<0.01). A total of 776 census tracts had >3 *E. coli* isolates collected during the study period and were included in the spatial and ecologic analysis of *E. coli* isolates. The average CTX-R percentage of *E. coli* isolates among census tracts was 9.6%. Autocorrelation analysis (Moran I) of CTX-R percentages among *E. coli* isolates also found a nonrandom distribution among census tracts (index 0.03, p<0.01). We mapped the geographic distribution of CTX-R percentages for all *Enterobacteriaceae* and for *E. coli* isolates alone (Figure 2).

We identified census tract–level characteristics reported in the 2017 American Community Survey of residents of the 886 census tracts that accounted for >3 *Enterobacteriaceae* isolates and compared census tracts with ceftriaxone resistance (461 [52.1%] of census tracts, mean 15.5 isolates/census tract) and without (425 [47.9%] of census tracts, mean 8.03 isolates/census tract). Bivariate analysis found that the presence of CTX-R isolates was negatively associated with census-tract percentages of non-Hispanic White and non-Hispanic Black populations, and positively associated with census-tract percentages of Hispanic, foreign-born, and uninsured residents. We observed no statistical associations between the outcome and percentages of households with incomes below the federal poverty level or with overcrowding (Table 2). Census tract-level characteristics were moderately correlated (r = −0.78 to 0.69).

**Table 2:** Population-level risk factors for ceftriaxone-resistant *Enterobacteriaceae* identified in Cook County census tracts, Illinois, USA, 2016–2018

Risk factor	Bivariate analysis	
	Odds ratio (95% CI)	p value
Non-Hispanic White population	0.98 (0.98–0.99)	<0.01
Non-Hispanic Black population	0.99 (0.99–0.99)	<0.01
Hispanic population	1.02 (1.01–1.02)	<0.01
Foreign-born population	1.02 (1.01–1.03)	<0.01
Households below poverty	1.00 (0.99–1.39)	0.35
Overcrowding	1.16 (0.97–1.39)	0.10
Uninsured population	1.07 (1.05–1.10)	<0.01

Among the 776 census tracts with >3 *E. coli* isolates, 395 (50.9%) had no CTX-R isolates and 381 (49.1%) had >1 resistant *E. coli* isolate collected during the study period, with an average CTX-R percentage of 19.4%. Bivariate analysis showed a negative association between presence of CTX-R *E. coli* isolates in census tracts and percentage of non-Hispanic Black population. Conversely, the odds of ceftriaxone resistance in an *E.coli* isolates was positively associated with the percentage of Hispanic, foreign-born, and uninsured residents and with residential overcrowding (Table 3).

**Table 3:** Population-level risk factors for ceftriaxone-resistant *Escherichia coli* identified in Cook County census tracts, Illinois, USA, 2016–2018

Risk factor	Bivariate analysis	
	Odds ratio (95% CI)	p value
Non-Hispanic White population	0.99 (0.99–1.00)	0.44
Non-Hispanic Black population	0.98 (0.98–0.99)	<0.01
Hispanic population	1.03 (1.02–1.03)	<0.01
Foreign-born population	1.04 (1.03–1.05)	<0.01
Households below poverty	0.99 (0.98–1.00)	0.24
Overcrowding	1.25 (1.04–1.53)	0.02
Uninsured population	1.08 (1.06–1.11)	<0.01

All 10,891 *Enterobacteriaceae* isolates (1,035 [9.5%] of which were CTX-R) were included in the individual risk analysis of patients from whom CTX-R and CTX-susceptible *Enterobacteriaceae* were recovered (Table 4). In the bivariate logistic regression analysis, male sex, an age range of 35–85 years, race and ethnicity other than non-Hispanic Black, and inpatient encounter were found to be associated with a higher likelihood of ceftriaxone resistance in a clinical isolate. Similarly, higher odds for the outcome were associated with the percentage of foreign-born residents in the census tract of isolate provenance.

**Table 4:** Individual and population-level risk factors for ceftriaxone-resistant *Enterobacteriaceae* in patients, Cook County Health healthcare system, Illinois, USA, 2016–2018*

Characteristic	No. (%) isolates				Bivariate analysis	
	All	Ceftriaxone-susceptible	Ceftriaxone-resistant		OR (95% CI)	p value
Total no. isolates	10,891 (100)	9,856 (90.5)	1,035 (9.5)			
Sex						
F	7,853 (72.1)	7,215 (66.2)	638 (5.9)		Referent	
M	3,038 (27.9)	2,641 (24.2)	397 (3.7)		1.7 (1.5–1.9)	<0.01
Age group, y						
18–34	2,011 (18.6)	1,895 (17.4)	116 (1.2)		Referent	
35–51	3,109 (28.5)	2,846 (26.1)	263 (2.4)		1.5 (1.2–1.9)	<0.01
52–68	4,092 (37.5)	3,667 (33.7)	425 (3.8)		1.9 (1.5–2.3)	<0.01
69–85	1,471 (13.5)	1,259 (11.6)	212 (1.9)		2.8 (2.2–3.5)	<0.01
>85	208 (1.9)	189 (90.9)	19 (9.1)		1.6 (0.9–2.7)	0.05
Race and ethnicity						
Non-Hispanic White	997 (9.2)	902 (8.3)	95 (0.9)		1.6 (1.2–2.0)	<0.01
Non-Hispanic Black	4,394 (40.4)	4,120 (37.8)	274 (2.6)		Referent	
Hispanic	4,898 (44.9)	4,324 (39.7)	574 (5.2)		1.9 (1.7–2.3)	<0.01
Other†	602 (5.5)	510 (4.7)	92 (0.8)		2.7 (2.1–3.5	<0.01
Encounter type						
Outpatient	5,889 (54.1)	5,419 (49.8)	470 (4.3)		Referent	
Emergency department	2,890 (26.5)	2,655 (24.4)	235 (2.1)		1.0 (0.9–1.2)	0.80
Inpatient	2,112 (19.4)	1,782 (16.4)	330 (3.0)		2.1 (1.8–2.5)	<0.01
Mean % foreign-born population (SD)‡	21.5 (17.0)	21.04 (17.0)	25.8 (16.2)		1.0 (1.0–1.1)	<0.01

*Values are no. (%) except as indicated. OR, odds ratio. †Other refers to isolates from participants who identified as non-Hispanic and reported race as Asian (3% of all isolates), American Indian/Alaska Native (0.4%), multiple races (0.1%), or unknown race (0.8%). ‡Based on data from 2017 American Community Survey 5-year estimates (*19*).

## Discussion

Our study has 4 main findings. First, compared with patients from whom CTX-susceptible community-onset *Enterobacteriaceae* isolates were collected, patients with CTX-R isolates more often were male, were 35–85 years of age, had self-identified race and ethnicity other than non-Hispanic Black, were hospitalized rather than discharged from the ED or seen in clinic, and resided in Cook County census tracts with higher proportions of foreign-born residents. Second, most patients with CTX-R isolates resided in a relatively small number of census tracts, with only 11% of *Enterobacteriaceae* isolate–generating census tracts accounting for 54.2% of CTX-R isolates and 93 (8.6%) of *E. coli* isolate–generating census tracts accounting for 49.7% of all CTX-R *E. coli* isolates. Third, spatial analysis supported the nonrandom distribution of Cook County census tracts generating higher proportions of ceftriaxone resistance among *Enterobacteriaceae* and *E. coli* isolates. Fourth, the population-level characteristics of census tracts from which isolates of CTX-R *Enterobacteriaceae* and *E. coli* were obtained differed from residents of census tracts yielding susceptible isolates exclusively, with the percentage of Hispanic residents, foreign-born, and uninsured population being positively associated with the presence of CTX-R isolates on analysis in both cohorts.

Similar to our findings, spatial studies conducted abroad of drug-resistant *Enterobacteriaceae* have shown nonrandom spatial distribution of antimicrobial-resistant *Enterobacteriaceae* in large urban areas. A study from São Paulo, Brazil (*20*), identified hotspot clusters of ciprofloxacin-resistant *E.coli* isolates that were associated with population-level ciprofloxacin usage. A study from Japan (*21*) also showed clustering of levofloxacin-resistant *E. coli* isolates in the western part of the country, also associated with population-level quinolone usage. In Chicago, residence in the northwest and southern region of Chicago (and adjacent suburbs) was independently associated with increased likelihood of infection by CTX-M-9 *Enterobacteriaceae* isolates in children (*22*).

Our individual-level analysis showing that ceftriaxone resistance was associated with increasing age and male sex is consistent with data reported elsewhere (*8*,*23*) and might reflect unmeasured associated underlying conditions, especially those involving the genitourinary tract (*8*,*24*) and antibiotic exposures (*8*,*25*–*27*). Unmeasured underlying conditions and associated antibiotic exposure could also account for the strong association between ceftriaxone resistance and the need for hospitalization, although the increased virulence observed in circulating ESBL-producing clones (*28*) could account for this finding.

The associations between self-reported Hispanic ethnicity and CTX-R *Enterobacteriaceae* and *E. coli* identified in the individual-level analysis and ecologic analyses merit further scrutiny. First, the correlation of Hispanic ethnicity and foreign-born status at a population level (r = 0.69) suggests that these 2 communities are highly interrelated; indeed, ≈45.6% of foreign-born persons in Cook County are noted to have emigrated from Latin America (*19*). Therefore, patients who self-identified as Hispanics also might have been foreign-born and might have become colonized by resistant organisms before emigration from or during travel to Latin American countries, some of which have reported high prevalence of ESBL-producing *Enterobacteriaceae* (*5*,*29*,*30*). This same pathway could explain the similar association between the proportion of foreign-born population in a census tract and likelihood of ceftriaxone-resistance in the ecologic and individual-level analyses. In addition, a sizable proportion of non–US-born Cook County residents emigrated from countries in Asia (27.3%) and fewer emigrated from Africa (3.2%) (*19*), continents with variable but often high prevalence of drug-resistant *Enterobacteriaceae* (*3*,*4*) (We did not include other racial and ethnic population-level characteristics in our individual-level analysis because of multicollinearity with individual-level race and ethnicity). Second, Hispanics residing in the United States have been reported to use antibiotics without prescription more frequently than other racial and ethnic groups (*31*). Third, we cannot discount that proximity of Hispanic communities, foreign-born communities, or both to environmental sources, such as contaminated waterways, might be an important added risk factor for colonization or infection by drug-resistant *Enterobacteriaceae* in these areas.

Although in our ecologic analysis the percentage of households beneath the poverty line was not significantly different between census tracts from which CTX-R *Enterobacteriaceae* or *E. coli* isolates were and were not generated, observed associations between the percentage of uninsured residents and the presence in census tracts of CTX-R isolates suggest that census tract-level deprivation might predispose to antimicrobial-resistant infections. In the analysis limited to *E. coli* isolates, overcrowding percentages were also associated with antimicrobial-resistant infections, suggesting possible household-level transmission. A recently published study by Otter et al. from London (*27*) identified associations between community-level variables, individual-level variables, and likelihood of ESBL rectal colonization among patients admitted to the hospital. In their analysis, only recent overseas travel, recent antimicrobial use, and community-level overcrowding rates were associated with ESBL rectal carriage, whereas individual- and community-level race, ethnicity, and immigration characteristics were not. The paucity of spatial and ecologic studies of antimicrobial-resistant *Enterobacteriaceae* in the United States makes it difficult to establish whether our results are representative of the urban epidemiology of these organisms in the country. Although not directly comparable because of a difference in outcomes, the discrepancy of our findings and those reported by Otter et al. (*27*) suggest that the effect of population-level variables might remain distinct in different geographic areas.

Our findings are limited by the fact that our isolates were obtained in a single healthcare system. As a safety-net healthcare system, CCH is likely to be subject to geographic bias already because our patients do not come equitably from all census tracts in Cook County. The paucity of isolates from Cook County communities that do not obtain services from our healthcare system limits the generalizability of our findings regionally. We were unable to gather data regarding risk factors for healthcare-associated infections (such as recent hospitalization) and recent antimicrobial use, both important limitations. In addition, the relatively small sample size and high correlation between population-level factors made meaningful multivariable analysis infeasible. We were unable to perform genomic analysis of CTX-R organisms, which would have enabled us to evaluate the relatedness of isolates and make stronger inferences about whether spatial clustering was related to a point source or interpersonal transmission. Finally, the limited number of clinical and population-level variables included in the individual risk analysis prevents definite conclusions regarding individual risk for CTX-R infection among our patients. Indeed, concurrent assessment of other well-known individual risk factors, such as recent travel or antimicrobial use, could alter the effect size of ecologic variables. Nevertheless, our findings corroborate previous investigations that have identified important community-level variation in CTX-R infection risk in association with geographic (*20*–*22*), demographic (*7*,*23*–*25*), and population-level variables (*27*). Developing effective mitigation strategies, such as focusing antimicrobial stewardship efforts on affected areas, including residence as a risk factor in treatment-decision algorithms, or identifying and eradicating local environmental sources of drug-resistant pathogens, could well depend on improved understanding of these dynamics.
